# Understanding how colorectal units achieve short length of stay: an interview survey among representative hospitals in England

**DOI:** 10.1186/s13037-014-0050-5

**Published:** 2015-01-23

**Authors:** Ben E Byrne, Anna Pinto, Paul Aylin, Alex Bottle, Omar D Faiz, Charles A Vincent

**Affiliations:** Imperial Patient Safety Translational Research Centre, Imperial College London, Office 5.03, 5th Floor, Medical School Building, St Mary’s Campus, Norfolk Place, London, W2 1PG UK; Dr Foster Unit, Department of Primary Care and Public Health, Imperial College London, London, UK; Surgical Epidemiology, Trials and Outcome Centre (SETOC), St Mark’s Hospital, Harrow, Middlesex UK; Department of Experimental Psychology, University of Oxford, Oxford, UK

**Keywords:** Colorectal surgery, Enhanced recovery, Length of stay, Outcomes, Interviews, Quality, Performance

## Abstract

**Background:**

Wide variation in the outcomes of colorectal surgery persists, despite a well-established evidence-base to inform clinical practice. This variation may be attributed to differences in quality of care, but we do not know what this means in practical terms of care delivery. This telephone interview study aimed to identify distinguishing characteristics in the organisation of care among colorectal units with the best length of stay results in England.

**Methods:**

Ten English National Health Service hospitals were identified with the shortest length of stay after elective colonic surgery between January 2011 and December 2012. Semi-structured telephone interviews were conducted with a senior colorectal surgeon and ward nurse, who were not informed of their performance, at each site. Audio recordings were professionally transcribed and thematically analysed for similarities and differences in practice between units.

**Results:**

All ten short length of stay units approached agreed to participate, and 19 of 20 interviews were recorded. These units standardised clinical care based upon an Enhanced Recovery Program. Beyond this, they organised the clinical team to efficiently and reliably deliver this package of care, with the majority of day-to-day care delivered by consultants and nurses. Patients were closely monitored for postoperative deterioration, using a combination of early warning scores, nurses’ clinical judgement and regular senior medical review. Of note, operative volume and laparoscopy rates in these units were not statistically significantly different from the national average (*p* = 0.509 and *p* = 0.131, respectively). The postoperative analgesic strategy varied widely between units, from routine epidural use to local anaesthetic infiltration or patient-controlled analgesia.

**Conclusions:**

The Enhanced Recovery Program may be seen as necessary but not sufficient to achieve the best length of stay results. In the study units, consultants and nurses led and delivered the majority of patient care on the ward. High quality teamwork helped detect and resolve clinical issues promptly, with nurses empowered to contact consultants directly if needed. Other units may learn from these teams by adopting protocol-based, consultant- or nurse-delivered care, and by improving coordination and communication between consultants and ward nurses.

**Electronic supplementary material:**

The online version of this article (doi:10.1186/s13037-014-0050-5) contains supplementary material, which is available to authorized users.

## Background

Arguably the biggest challenge facing health care at present is not deciding what we should do for our patients, but how to make sure we reliably and efficiently deliver care in a way that we already know results in good outcomes. In the specialty of colorectal surgery, a significant body of evidence has developed in favour of Enhanced Recovery Programs [1-3] and laparoscopic surgery [4-6]. Yet even when optimal management is widely known and understood, wide variation in the outcomes of colorectal [7-9] and other forms of surgery [10-12] have been repeatedly demonstrated. This variation has been attributed to differences in the quality of care of different providers [13-16]. However, we do not know what this means in practical terms. What is it that 'high quality' providers do differently in their day-to-day management of patients and organisation of care?

The approach to understanding performance adopted in this study has much in common with the 'positive deviance' methodology described by Bradley et al. [17]. This involves identifying high performing teams using accepted measures of performance that demonstrate variation between organisations. Units are studied using qualitative methods to understand determinants of performance, which may then form the basis of quantitative assessment and quality improvement. This approach has been used to understand and improve door-to-balloon time in acute myocardial infarction [17-19], as well as time to thrombolysis after stroke [20].

We present the qualitative analysis of twenty semi-structured telephone interviews with surgeons and nurses at English colorectal units, selected for having excellent length of stay results. The study aimed to identify similarities in care across these high performing units that may have been key in achieving their results.

## Methods

### Cohort selection

The Dr Foster Unit at Imperial College and its commercial partner, Dr Foster Intelligence, routinely process administrative Hospital Episode Statistics (HES) data from the English National Health Service (NHS) [21]. Risk-adjusted measures derived include the frequency of long length of stay (LLoS), defined as any in-patient stay that exceeds the 75th centile for the national cohort. Ten hospitals each were selected for interview if they had above or below average risk-adjusted LLoS for adult patients undergoing elective colonic surgery between January 2011 and December 2012. Poor engagement of high LLoS units precluded meaningful analysis of this group's results. Therefore, this paper presents the results of interviews with hospitals with low frequencies of LLoS.

### Interviews

The interview schedule was designed to obtain a broad overview of the factors that may be associated with institutional outcomes in elective colonic surgery. The interview was framed within Donabedian's structures, processes and outcomes framework [22,23]. A consideration of the patient journey, with pre-, intra- and post-operative phases was used, together with an established framework for analysing risk and safety in medicine [24]. The resulting preliminary protocol was refined in the light of pragmatic literature searches for relevant evidence, as well as piloting with local experts and healthcare researchers. The final protocol covered 11 key areas (see Table 1).

**Table 1:** **Interview themes**

**Domain**	**Factor**
Structure	Equipment
	Staffing
Process - clinical	Pre-operative assessment
	Operative details
	Routine postoperative management
	Detection and management of complications
Process - institutional	Standardisation
	Communication and collaboration
	Leadership and culture
	Attitudes to safety and adverse events
	Outcomes assessment and feedback

Areas of care organisation covered in the interview.

Semi-structured telephone interviews were conducted with a senior colorectal surgeon and a senior colorectal ward nurse or sister in each hospital. Interviewees were not informed of their unit's performance. Participants provided written consent. Interviewees were asked open scripted questions, and prompts and probes were not pre-specified. All interviews were conducted by BEB, a general surgical trainee. Interviews were completed during July and August 2013, typically lasting 30 to 40 minutes. No repeat interviews were required. Interviews were audio recorded and professionally transcribed verbatim.

During the interview, participants were asked to estimate laparoscopy rates. Laparoscopy rates were also ascertained from in-house HES data relating to surgery between January 2011 and March 2012.

### Data analysis

Thematic qualitative analysis of interview transcripts [25] was performed to identify similarities and differences in the organisation and delivery of care between the study units. Initial analysis was performed by coding the detail of each transcript. Intermediate and higher level categories were developed as connections emerged between coded sections. This resulted in a hierarchical tree of super- and sub-ordinate themes. Initial coding was performed independently by BEB and AP on three randomly selected interviews. After meeting to review emerging themes, two further interviews were independently coded. A further meeting to review and consolidate codes was convened. There was good agreement between researchers at both meetings. All remaining analyses were completed by BEB. Coding was performed using NVivo 10 for Windows (QSR International Pty Ltd, Melbourne, Australia).

### Ethical approval

The Imperial College London and Imperial College Healthcare NHS Trust Joint Research Compliance Office advised that the interviews met criteria as a service evaluation and were exempt from ethical review. Local project approval processes were followed at participating hospitals. The authors have permission from the Confidentiality Advisory Group under Section 251 of the NHS Act 2006 (formerly Section 60 approval from the Patient Information Advisory Group) to hold confidential NHS data and analyse them for research purposes (PIAG 2-05(d)/2007). The authors also hold ethical approval for such work from the South East Research Ethics Committee (10/H1102/25).

## Results

All ten low LLoS units approached took part, and 19 of 20 interviewees consented to audio recording of the interview. LLoS results and other descriptive characteristics are provided in Table 2. Study units did not statistically significantly differ from non-study units in terms of caseload or laparoscopy rates (independent samples t-test, equal variances not assumed, *p* = 0.509 and *p* = 0.131, respectively).

**Table 2:** **Unit characteristics**

**Site code**	**Long length of stay**		**Caseload** ^*^	**Number of colorectal consultants** ^**±**^	**ERP formally introduced**	**Laparoscopy rates (%)**	
	**Risk-adjusted frequency**	**95% CI**				**Consultant estimate** ^**+**^	**HES derived** ^**¥**^
1	0.24	0.10-0.46	158	3	2011	50	46
2	0.32	0.10-0.74	68	2.2	2010	70	58
3	0.48	0.23-0.89	91	3	2008	49	47
4	0.50	0.30-0.77	193	5	2006	85	85
5	0.34	0.13-0.69	99	4	2007	80	79
6	0.38	0.15-0.78	91	4	2013	70	77
7	0.38	0.14-0.84	75	4	2008	60	48
8	0.49	0.33-0.71	261	6	N/A	33	31
9	0.42	0.22-0.73	137	3.5	2008	60	59
10	0.47	0.25-0.81	129	3	2011	40	36
Study av.	0.40	-	130	3.8	-	60	57
National av.	0.97	-	118	-	-	-	47

Summary characteristics and Long Length of Stay results of participating units. CI – Confidence Interval; HES – Hospital Episode Statistics; av. - average; ^*^ - elective colonic surgery between January 2011 and December 2012; ^±^ - whole time equivalents; ^+^ - between July and August 2013; ^¥^ - between January 2011 and March 2012.

Qualitative thematic analysis identified three key themes in the organisation of care across the units included in the study, as described below.

### Define and standardise clinical processes

Nine of the ten study hospitals had adopted a formalised patient pathway based upon the ERP, with the tenth having piloted the ERP and adopted many components of this care package. Accordingly, the units standardised a number of clinical processes relating to the pre-, intra- and post-operative phases of the patient journey. The study was not designed to comprehensively assess all details of patient care. Nonetheless, extensive preoperative counselling, and postoperative mobilisation and oral intake were of clear importance across all study units (see Additional file 1: quotation box 1).

While practice within units was largely uniform, some areas of care demonstrated wide variation between different units. Consultant-estimated laparoscopy rates ranged from 33% to 85%, and postoperative analgesic strategies varied from routine epidural usage, to active avoidance of epidurals, use of local anaesthetic infiltration devices or patient controlled opiate analgesia.

### Organise team to deliver care reliably and efficiently

Across the study units, consultants and nurses adopted a lead role in direct care provision on a day-to-day basis. There was less reliance on medical trainees to lead clinical care. Appropriately trained nurses undertook preoperative patient counselling and risk assessment, and, in some hospitals, led postoperative ward care (see Additional file 1: quotation box 2). Such nurse-led care required close consultant support. Other hospitals described how consultant presence on the ward was required to maintain efficient patient flow along the pathway (see Additional file 1: quotation box 2).

Besides the allocation of clinical tasks to specific members of the team, the majority of hospitals in the study organised patients on wards by specialty or urgency of admission.

### Monitor and respond to deviations from the norm

At every hospital site, nurses used an observation-based early warning score system, with associated protocols for escalation of care, to detect deterioration in their patients. Beyond this, a number of nurses described a more sophisticated approach, using patients' symptoms and signs and previous clinical experience, to help detect complications before physiological deterioration (see Additional file 1: quotation box 3). Across the studied units, several nurses described reporting concerns directly to consultants, bypassing more traditional patterns of escalation within the medical team (see Additional file 1: Box 4). This relied on excellent relationships between nurses and consultants, with open communication and respect for nurses' opinions and judgements.

The study sites also reported frequent senior medical input. Most consultants saw their patients from 2 to 3 times per week, to every day. In all but one site, the daily ward round was conducted by at least a registrar, if not a consultant. Some sites reported routine review two or three times per day.

Resources for responding to complications differed between the study sites. All had access to emergency theatres, intensive care and interventional radiology, but the level of support varied. Emergency theatres were often shared between specialties. A number of interviewees commented that while the intensive care team were very supportive and helpful, they were often stretched. Some sites had 24-hour access to interventional radiology through an on-call system, while others only had routine access during normal working hours.

## Discussion

This study suggests that a program of perioperative care based upon the ERP may be a key pre-requisite for achieving excellent length of stay results. However, the delivery of this care package in day-to-day practice on the ward was critically important. To achieve the shortest length of stay, the study units organised care to ensure patients were managed consistently and efficiently in accordance with the local protocol, with much direct clinical care delivered by nurses and consultants. In addition, postoperative patients were carefully monitored for deterioration, using a combination of early warning scores, empowerment of nurses to exercise their clinical judgement, and frequent senior medical review. Excellent inter-professional communication facilitated early consultant involvement in the management and resolution of problems. It is of interest that the study units had similar laparoscopy rates to non-study units across the country. Within the study, there was marked variation in laparoscopy rates and postoperative analgesic approaches between units. These observations suggest that while these factors may be important determinants of outcome within the confines of a clinical trial, other factors may be equally important in ‘real-world’ clinical practice.

This study has several strengths. LLoS was derived from administrative data, which represent a population-based information source. Therefore reporting bias, as found in voluntary disease registers [26,27], will have been minimised. The accuracy of administrative data for outcomes research may be questioned, but its validity is increasingly accepted and backed by research [28,29]. All interviews were conducted by BEB, a surgical trainee. This helped establish rapport with interviewees, who provided rich opinions in relatively short interviews. Double-coding of the first 5 interviews with a non-clinical health care researcher (AP), who has extensive experience with qualitative methods, ensured the rigor of this analysis.

The study also has a number of limitations. The units included represent a small sample of the population of colorectal units, and further work is planned to develop and confirm these initial findings in units across a range of performance. Interview data may be subject to social desirability response bias, where participants alter how they represent their work in response to the social pressures and context of such an interview. While interviewees were not informed of their organisation’s performance, their description of local practice may have been positively biased. Only one surgeon and one nurse were interviewed at each site; different data may have been gathered from other members of the same organisations. The data collected on length of stay used to select units for interview pertained to a period ending over six months before the interviews were conducted. However, updated LLoS results between January 2012 and December 2013 showed that all units continued to have lower than average LLoS rates, with nine of the ten units having upper 95% confidence intervals below 1.0.

Few studies have been conducted using similar techniques to understand high performing institutions. Bradley et al. [18,19] studied American hospitals achieving short door-to-balloon times for patients admitted with acute ST-elevation myocardial infarctions. This involved 11 site visits, and in-depth interviews lasting 1 to 1.5 hours with 122 staff. Their qualitative methods were more rigorous, yet the themes identified were similar to the present study. For example, 2 themes related to standardised protocols, and one to collaborative teamwork [19]. A smaller study of door-to-needle times for stroke thrombolysis identified similar themes [20]. Comparable results between these earlier studies and the presented work support the validity of the methodology, data and analysis used in this study. Semi-structured telephone interviews represent a cheaper and quicker source of data than site visits and in-depth interviews.

This study highlights the importance of organisational factors in determining high performance. The clinical care provided by the study institutions was founded upon well-known, evidence-based practices within colorectal surgery. The ERP was necessary but not sufficient to achieve the best results. Beyond the ERP, the units all carefully managed how this care package was actually delivered and implemented.

*‘…All the obvious stuff for this [reducing length of stay] has been said and proven a million times before. Enhanced Recovery does work, laparoscopic surgery does work, goal-driven senior management does work … so all these things are fairly obvious … Next stage I think now is to get people to actually follow the things that you know work’.*

Site 2, surgeon

In this study, ward nurses had a key role in complication detection and management. Excellent inter-professional communication was developed through frequent and senior medical presence on the ward. Strong relationships helped empower nurses to seek definitive senior assessment and treatment for their patients earlier, bypassing intermediary assessments by more junior team members. Experience managing routine care may have helped nurses to better discriminate normal and abnormal recovery. Furthermore, pattern learning may have been simplified and accelerated by managing all patients similarly on the same ward. The important contribution of nurses in these areas is supported by meta-analytic evidence of an association between increased registered nurse staffing and lower mortality and failure to rescue rates in surgical patients [30].

Increasing reliance on consultants and nurses to deliver care represents a paradigm shift in the organisation of the clinical team. Though evidence supporting improved outcomes with consultant-led or -supervised care in the operating theatre is mixed [31-33], some research suggests consultant-delivered ward care improves results [34-36]. Such a shift in workload may be an excellent strategy for improving the quality of care, but it may meet resistance from certain stakeholders. Arguments against such a shift include increased demands being placed on already stretched members of the team, and de-skilling of medical trainees. If consultants and nurses are to take on greater responsibility for direct care delivery, this needs appropriate support and staffing. While consultant- and nurse-delivered care may take some responsibilities away from trainees, it may also represent an improved opportunity for skills and knowledge transfer from experienced staff to trainees. It may also free trainees from certain service commitments to focus more time on structured training.

Potential disadvantages of such changes should be weighed against their advantages. Stability and consistency of care over time may improve both efficiency and safety. Increased efficiency may result in reduced length of stay and lower costs. Reduced variation, improved continuity and closer working relationships between consultants and nurses may reduce errors and improve the early detection and resolution of problems. These may indirectly reduce the costs of care. From a patient's perspective, there are no negatives from such a shift in the organisation of care.

This paper highlights the merits of exploring novel or under-utilised methods for the study of health care performance. While much has been learned by analysing poor institutional performance, the recurrence of scandals within the NHS, such as the Bristol Royal Infirmary Inquiry [37] and Mid Staffordshire Inquiries [38,39], suggests that this approach has not delivered the intended improvements in care. Perhaps change may be achieved by providing a positive vision for health care, using lessons learned from peers who have achieved excellent results within the same system. Future research using similar methods may help clarify whether similar factors are associated with high performance defined using other outcomes, and in other fields of medicine.

## Conclusions

The colorectal units in this study achieved excellent length of stay results, founded upon the ERP. However, an ERP alone may not be enough. The majority of direct clinical care was delivered and led by consultants and nurses, resulting in efficient delivery of the ERP and excellent organisational outcomes. Well-supported nursing staff and frequent medical review helped identify and resolve care problems promptly. Laparoscopy rates were not significantly different from the national average. An appropriately supported shift in responsibility for care delivery towards nurses and consultants, and away from trainees, may be an effective strategy for the improvement of colorectal outcomes, though further work is required to generalise the study findings more widely.

## Additional file

Additional file 1:
**Selected quotes from interview transcripts.**

## Abbreviations

NHS: National Health Service
ERAS: Enhanced recovery after surgery
ERP: Enhanced recovery protocol
LLoS: Long length of stay
HES: Hospital Episode Statistics
