# Supplementary material for: Understanding how colorectal units achieve short length of stay: an interview survey among representative hospitals in England
Source: Patient Saf Surg. 2015 Jan 23;9:2. doi: 10.1186/s13037-014-0050-5 (PMC4304175; doi:10.1186/s13037-014-0050-5)
Supplement: Additional file 1: — Selected quotes from interview transcripts. [file 13037_2014_50_MOESM1_ESM.docx]

**Quotation boxes**

Box 1. Quotes describing the importance of the Enhanced Recovery Program and pre-operative counselling, one of its key components.

| *'I think it's [the ERP is] great for the nursing staff, because they know where they are, [and] what they need to be doing with their patients, which is really good.'*  Site 6, nurse |
| --- |
| *‘I think it [the ERP] works really well … because it helps every single member of staff and it helps the patient because … they know what is expected of them …’*  Site 1, nurse |
| *‘...It's been our experience that one of the big barriers for patients going through an Enhanced Recovery Program is pre-defined concepts about what they're going through, and it's impossible to have a colonic resection and be out of hospital within two days, "My dad was in for a week," etc. So we're very keen to sow the seeds of early discharge, if all goes well, right from the start.'*  Site 9, surgeon |

Box 2. Quotes describing nurse and consultant leadership of routine care.

| *'...The Enhanced Recovery ... gives us the wherewithal to be a sort of nurse-led sort of protocol. We have a protocol set by all three consultants, but we sort of dictate when lines are taken out, when people start to drink, etc, etc.'*  Site 1, nurse |
| --- |
| *'Nurses are far better at following protocol than doctors are, and they tend to react better... doctors think they know better, if you see what I mean? So if you have nurse-led discharge, if you have nurse-led Enhanced Recovery Programs, then things tend to happen in a much more structured fashion than if you leave it up to the doctors.'*  Site 2, surgeon |
| *'...The issue I have with the ERP ... [is] unless we have a consultant actually seeing the patient daily and driving it forward, they [patients] tend to get delayed in their discharge. So, actually, it's consultant-based care and ward management.'*  Site 3, surgeon |

Box 3. Quotes describing the role of nurses in the detection of postoperative complications.

| *'...From an experienced nurse perspective, you just know when there is something wrong. ... You don't have to particularly look at the NEWS [early warning score] shot, you can look at a patient and sort of see clinically they're not quite right. ... Then obviously we'd inform whoever we felt was appropriate depending upon what their NEWS result was.'*  Site 1, nurse |
| --- |
| *'In this Trust we have a PAR [early warning score] scoring system, so all our patients have four-hourly observations performed. Within that we also have a two-hourly rounding program. ... So it's normally either within the observation round or through the two-hourly rounding that problems can arise, for example, if someone hasn't passed wind, hasn't been out to the toilet post bowel surgery, that would be triggered when you're asking your rounding questions. So it's normally at that point that I write, 'He might have an ileus.' You'd let the team know, listen to bowel sounds, etc.'*  Site 4, nurse |
| *'The nursing staff will communicate any concerns, worries, directly to the consultant ... Most importantly, they don't have to reach physiological parameters for a nurse to be allowed to phone the consultant, in the hope that the nursing staff spot that someone is unwell before they become physiologically unwell.'*  Site 5, surgeon |

| *'We've got the Early Warning Score that we use, so if a patient scores anything above a three ... we escalate it, and that goes to the F1, reg. But, with the Enhanced Recovery, we tend to side-step them and go straight to the consultant.'*  Site 5, nurse |
| --- |
| *‘If there’s any deviation [postoperatively] or things aren’t going as planned, then obviously the relevant consultants tend to get called and phoned anyway, as a rule.’*  Site 4, surgeon |
| *'...We all work together as a team, and any member of my staff could contact any of the consultants at home and would feel happy to do that. And I would do that without hesitation if I felt that a patient was being mis-managed, and I have done in the past.'*  Site 1, nurse |

Box 4. Quotes describing the direct escalation of nurses' concerns to consultants.
